# Recombinant production of plant lectins in microbial systems for biomedical application – the frutalin case study

**DOI:** 10.3389/fpls.2014.00390

**Published:** 2014-08-08

**Authors:** Carla Oliveira, José A. Teixeira, Lucília Domingues

**Affiliations:** Centre of Biological Engineering, University of MinhoBraga, Portugal

**Keywords:** recombinant frutalin, lectin isoforms, *Pichia pastoris* expression system, glycosylation, *Escherichia coli* expression system, biomedical application, tumor biomarker, apoptosis-inducer

## Abstract

Frutalin is a homotetrameric partly glycosylated α-D-galactose-binding lectin of biomedical interest from *Artocarpus incisa* (breadfruit) seeds, belonging to the jacalin-related lectins family. As other plant lectins, frutalin is a heterogeneous mixture of several isoforms possibly with distinct biological activities. The main problem of using such lectins as biomedical tools is that “batch-to-batch” variation in isoforms content may lead to inconstant results. The production of lectins by recombinant means has the advantage of obtaining high amounts of proteins with defined amino-acid sequences and more precise properties. In this mini review, we provide the strategies followed to produce two different forms of frutalin in two different microbial systems: *Escherichia coli* and *Pichia pastoris*. The processing and functional properties of the recombinant frutalin obtained from these hosts are compared to those of frutalin extracted from breadfruit. Emphasis is given particularly to recombinant frutalin produced in *P. pastoris*, which showed a remarkable capacity as biomarker of human prostate cancer and as apoptosis-inducer of cancer cells. Recombinant frutalin production opens perspectives for its development as a new tool in human medicine.

## OUTLINE

Plant lectins have attracted much attention for biomedical applications, especially due to their remarkable anti-tumor properties, resulting from their ability to induce programmed cell death and/or autophagocytosis in cancer cells (Liu et al., 2010; Fu et al., 2011). Plant lectins are also relevant for biomedical diagnosis (Mislovicova et al., 2009).

Frutalin is a plant lectin with reported immunomodulatory (Brando-Lima et al., 2005, 2006), anti-tumor (Oliveira et al., 2011), and tumor biomarker (Oliveira et al., 2009b) properties, among other capacities (de Vasconcellos Abdon et al., 2012), and is a good example of how recombinant production of plant lectins can be challenging but also advantageous for obtaining bioactive derivatives for biomedical application. Frutalin is found in extracts of *Artocarpus incisa* (breadfruit) seeds (Pineau et al., 1990), from which it can be purified by affinity chromatography on cross-linked *Adenanthera pavonina* galactomannan (Moreira et al., 1998). The name “frutalin” (hereinafter referred to as FTL) is a composite of part of the Portuguese common name of the lectin source plant (“fruta” of “fruta-pão”) followed by the suffix “-lin”. Although having sugar-binding preference toward D-galactose, FTL presents a rather broad sugar-binding activity, interacting also with other sugars, as D-mannose and D-glucose. FTL belongs to the jacalin-related lectins family (JRLs, found in the Moraceae plant family), specifically to the sub-family of the galactose-specific lectins (gJRLs), as it presents high structural homology, sugar specificity and sequential identity with jacalin (the galactose-specific lectin of *Artocarpus integrifolia* seeds – jackfruit, the first member of this family to be identified; Pineau et al., 1990; Moreira et al., 1998; Oliveira et al., 2009a). FTL is characterized by a strong and identical agglutinating activity with human erythrocytes of the ABO system and rabbit erythrocytes, which has no requirements for divalent metal cations (Moreira et al., 1998). Interestingly, the hemagglutination activity (HA) of FTL is three times higher than that of jacalin (Nobre et al., 2010). FTL has a sophisticated processing. The conversion of the primary translation product of gJRL-mRNA into the protein includes a complex series of co- and post-translational modifications including the removal of the signal peptide (vacuolar targeting), a (partial) glycosylation, removal of the *N*-terminal propeptide, the excision of a linker tetrapeptide, to separate two polypeptide chains (α and β), proper folding and oligomer assembly. Molecular cloning of the FTL cDNA (excluding signal and propeptide), revealed that, as jacalin, it may be encoded by a family of genes, each of them containing 471 bp, corresponding to a protein of 157 amino-acids, with a calculated molecular weight of 17.1 kDa (Oliveira et al., 2009a). Twenty amino-acids correspond to the β-chain, 4 amino-acids to the linker “T-S-S-N” and 133 amino-acids correspond to the α-chain (from *N*- to *C*-terminal). Several gJRLs conserved regions of amino-acids were found in FTL sequences, including the linker. The linker, and its processing, is specific for the sub-group of the gJRLs, being absent in the other sub-group (mannose-specific JRLs; Houles Astoul et al., 2002). FTL is a heterogeneous mixture of several slightly different amino-acid sequences sharing 93–97% of identity, with or without consensus sequences for *N*-glycosylation (Asn-X-Thr/Ser) in the α-chain (one of these potential *N*-glycosylation sites was also reported for jacalin; Oliveira et al., 2009a). In fact, FTL is a partly glycosylated protein, with 2.1% of carbohydrates (Moreira et al., 1998). Different FTL isoforms or iso-lectins (i.e., different mature sequences) may have distinct biological activities, as reported for other plant lectins (Raemaekers et al., 1999; Ohba et al., 2003). Under denaturing conditions (SDS-PAGE), FTL presents two bands: the upper band (15.5 kDa) corresponds to the highly glycosylated isoforms of α chain, whereas the lower band (12 kDa) represents the slightly or non-glycosylated isoforms of the same chain (Oliveira et al., 2008). The β chain is not visible due to its low molecular weight (2.1 kDa; Oliveira et al., 2008). In its native form, FTL is a tetrameric molecule, consisting of four monomers bound by non-covalent linkages, each containing one β and one α chain, forming four sugar-binding sites, with a predominantly β sheet conformation (Moreira et al., 1998; Campana et al., 2002) and an apparent molecular mass of 48–49 kDa (Moreira et al., 1998; Oliveira et al., 2008). FTL is a robust protein as it is stable up to 60°C and very resistant to chemical denaturation (Campana et al., 2002).

Plant lectins are commonly isolated from their natural sources, although this presents several disadvantages, as the resulting isoforms. Recombinant production, mainly in microbial hosts, is an interesting way to overcome this problem, whilst it may allow to improve availability, ensure continuous supply and facilitate purification of lectins with interesting activities or improved/tailor-made functionalities, particularly for biomedical application (for a recent review see Oliveira et al., 2013).

This review describes the case study of the different strategies applied for the production of FTL in the bacterium *Escherichia coli* (Oliveira et al., 2009a; Costa, 2013; Costa et al., 2013a) and in the yeast *Pichia pastoris* (Oliveira et al., 2008). Several variables were considered for optimization: codon usage, strains, fusion partners, induction conditions, and purification methodology. Both microorganisms are well-established platforms for the production of recombinant proteins, including several approved biopharmaceutical products (Berlec and Strukelj, 2013; Gasser et al., 2013). These are also the most employed hosts for the production of recombinant lectins, namely plant lectins for biomedical purposes, such as jacalin (Sahasrabuddhe et al., 2004, 2006), aviscumin (from *Viscum album*; Zwierzina et al., 2011), PCL (from *Polygonatum cyrtonema*; Li et al., 2011), Orysata (from *Oryza sativa*; Al Atalah et al., 2011), and GNA_maize_ (from *Galanthus nivalis*; Fouquaert et al., 2009). *E. coli* is commonly used to produce non-glycosylated lectins, while *P. pastoris* is mainly employed to overcome problems of insoluble expression of the bacterial system and to produce glycosylated lectins. Thus, *E. coli* and *P. pastoris* were chosen to produce non-glycosylated recombinant frutalin (EcrFTL) and glycosylated recombinant frutalin (PprFTL), respectively. The bio-molecular characterization of the recombinant FTL obtained from each host in terms of processing, molecular weight, HA and sugar-binding activity is herein presented. Finally, a main focus is given to PprFTL due to its demonstrated anti-tumor and tumor biomarker activities (Oliveira et al., 2009b, 2011).

## PRODUCTION OF RECOMBINANT FRUTALIN IN *E. coli*

**FIGURE 1:**
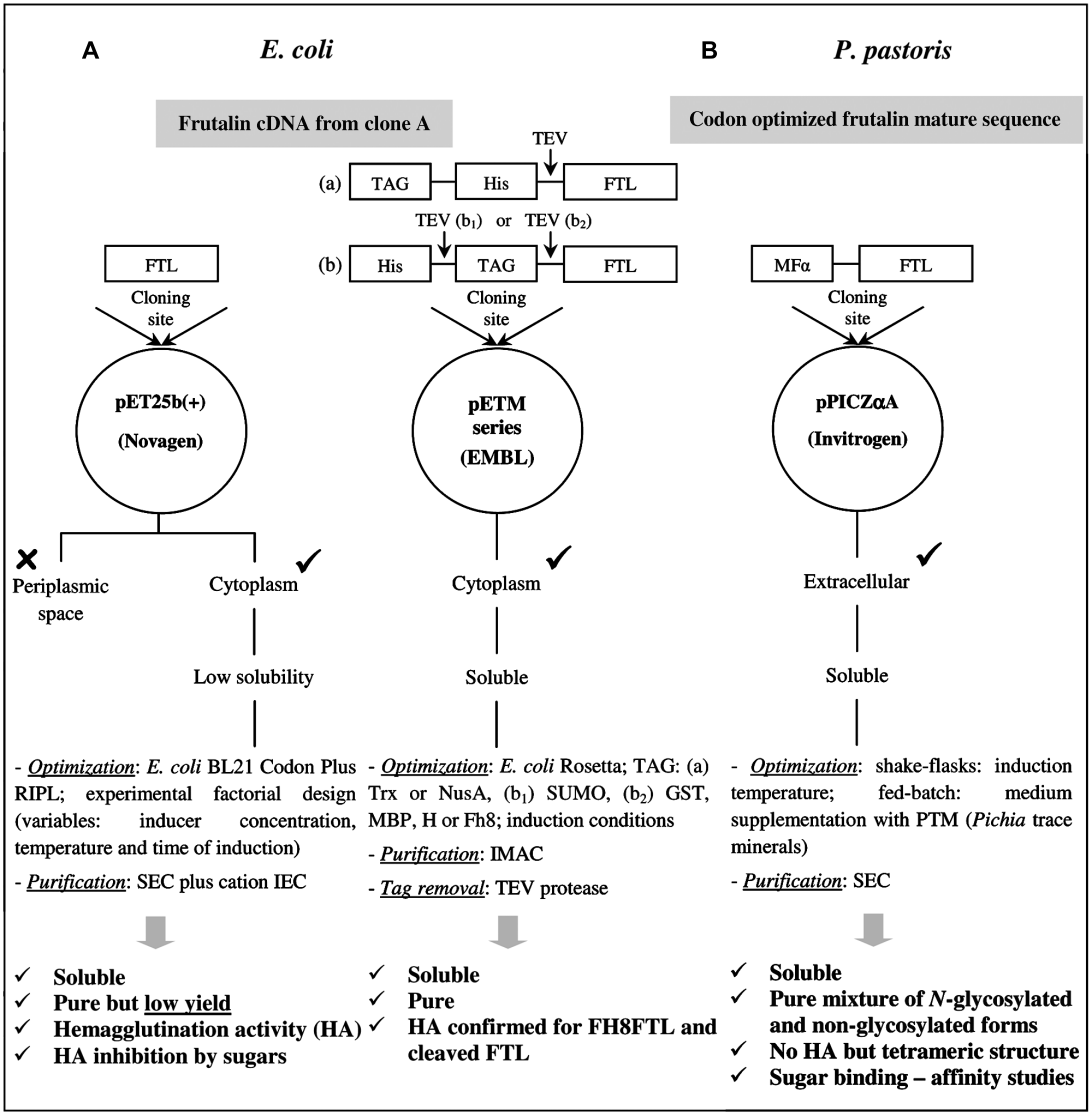
**Main strategies for the production of soluble recombinant FTL in *Escherichia coli* (A) and *Pichia pastoris*(B).** Different frutalin codifying genes were cloned in *E. coli* and *P. pastoris*, which deduced amino-acid sequences share 93% of identity (Table 2 in Oliveira et al., 2009a).

A FTL cDNA sequence was used for production of recombinant FTL in *E. coli* by different strategies (Oliveira et al., 2009a). The first attempts to produce soluble EcrFTL in *E. coli* focused in the use of engineered *E. coli* strains that have extra copies of rare tRNAs and in the optimization of the induction conditions, but resulted in low yields (Oliveira et al., 2009a; Costa, 2013). The soluble production of EcrFTL from strain *E. coli* BL21 Codon Plus RIPL (DE3), harboring the pET-25b(+) expression vector (Novagen), was maximized to 16 mg/l by the implementation of an experimental factorial design (Oliveira et al., 2009a; **Figure 1**). However, all the experimental conditions resulted in EcrFTL produced predominantly as insoluble protein. Even though, EcrFTL was purified from crude *E. coli* extracts by sequential size exclusion (SEC) and cation ion exchange chromatography (IEC) that yielded 76 μg of protein per liter of *E. coli* culture. Purified EcrFTL migrated in SDS-PAGE gel as a homogeneous single-band protein with a molecular mass of about 17 kDa, indicating that the linker was not cleaved. Nevertheless, EcrFTL presented HA against rabbit erythrocytes, although it required more time to develop this activity than FTL. Thus, the HA of FTL is not strictly dependent on linker cleavage. In assays of HA inhibition by different sugars, EcrFTL presented specificity for galactose; however, it could not be purified by affinity chromatography on *A. pavonina* galactomannan, thus revealing lower sugar-binding affinity than FTL. The biomedical properties of this EcrFTL were not evaluated since the amounts obtained through this strategy were unsatisfactory and we were willing to improve them.

Taking into account the low production yields previously obtained, fusion protein technology was afterward considered to improve the soluble production and purification of recombinant FTL in *E. coli*. Eight fusion tags (His_6_, Trx, GST, NusA, MBP, SUMO, H, and Fh8), included in pETM vectors (EMBL), were evaluated in small-scale screening assays for recombinant FTL solubility in four *E. coli* strains (Costa et al., 2013a; **Figure 1**). All vectors provided a His_6_ tag for purification of the EcrFTL fusions by nickel affinity (immobilized metal ion affinity chromatography – IMAC). The Rosetta strain (DE3) was selected for scale-up protein processing, namely purification and solubility evaluation before and after tag cleavage. The solubility enhancer partners NusA, Trx, and Fh8 tags considerably improved the soluble production of EcrFTL (in the order: NusA∼Fh8 > Trx), being the protein soluble after their removal by TEV cleavage (Tobacco Etch Virus protease). Interestingly, the cleaved and purified EcrFTL from the Fh8 and Trx fusions presented higher amounts than that cleaved from the NusA fusion protein (Costa et al., 2013a). The fusion protein strategy boosted the availability of EcrFTL by increasing its yield from μg to mg of active protein per liter of *E. coli* culture whilst simplifying the complete production and purification protocol (Costa, 2013). IMAC revealed to be a simpler, easier and quicker procedure than SEC or IEC for EcrFTL purification, and it also decreased EcrFTL losses during purification. Moreover, EcrFTL kept its HA when fused to this partner (our unpublished results). However, the His_6_FTL fusion (produced from vector pETM-11) was found to be a very unstable protein, precipitating at physiological pH, and thus being incompatible with cell culture conditions for evaluation of its anti-tumor activity (our unpublished results). Among the fusion partners tested, the Fh8 tag was pointed as a good option for the production of soluble EcrFTL in *E. coli* because of its advantageous low molecular weight and combined solubility enhancer and purification handle activities (Costa, 2013). The Fh8 tag (*Fasciola hepatica* 8-kDa antigen) has been recently ranked among the best solubility enhancer partners for recombinant protein production in *E. coli* (Costa et al., 2013a, 2014). Fh8 was also shown as a suitable fusion partner for purification of recombinant proteins in *E. coli* by HIC (hydrophobic interaction chromatography), with efficiencies comparable to those of IMAC (Costa et al., 2013b). Besides improving EcrFTL solubility, the Fh8 tag increased EcrFTL stability, compared to the His_6_ tag, and it did not interfere with the HA and structure (in respect to β-sheet conformation) of EcrFTL, before and after its cleavage (Costa, 2013). The anti-tumor activity of these two versions of EcrFTL, the Fh8FTL and Fh8-cleaved FTL, is under evaluation.

## PRODUCTION OF RECOMBINANT FRUTALIN IN *P. pastoris*

### PRODUCTION AND BIO-MOLECULAR CHARACTERIZATION

Frutalin has highly glycosylated isoforms and the presence of the glycans may be important for its functional properties. Thus, we planned to produce FTL in a microorganism capable of performing glycosylation, namely using the strain *P. pastoris* KM71H (Oliveira et al., 2008; **Figure 1**). FTL gene (encoding a mature sequence; Oliveira et al., 2009a) was optimized based on the codon usage of *P. pastoris* and integrated into the yeast genome *in frame* at the *C*-terminal of the *Saccharomyces* α-factor preprosequence (MFα), to direct PprFTL into the secretory pathway, and under the control of the strong methanol inducible *AOX*1 promoter. PprFTL was produced in shake-flasks and purified from supernatants by SEC yielding 18–20 mg per liter of culture.

Important differences between the molecular and biological properties of PprFTL and FTL were found (Oliveira et al., 2008). The reason for that was the processing of FTL in *P. pastoris*, which was different from that occurring in breadfruit. As also observed in *E. coli* (Oliveira et al., 2009a), the FTL linker was not cleaved in *P. pastoris* (confirmed by *N*-terminal sequencing), thus suggesting that this processing can be specific for higher eukaryotes. Furthermore, the MFα secretion leader was incompletely removed, resulting in PprFTL with one Glu-Ala repeat at its *N*-terminal, decreasing its predicted PI (isoeletric point) from 8 to 5. These repeats are commonly observed in heterologous proteins secreted by *P. pastoris* using this signal sequence, and also reported for other recombinant plant lectins (Raemaekers et al., 1999; Lannoo et al., 2007).

As expected, PprFTL was *N*-glycosylated by *P. pastoris*, since the corresponding protein sequence has one potential site for *N*-glycosylation (α-Asn74). Part of the secreted PprFTL undergone this post-translational modification, which led to an extension in its molecular weight of about 2.8 kDa (Oliveira et al., 2011). PprFTL, contrarily to FTL, did not agglutinate rabbit erythrocytes, despite also having a tetrameric structure (Oliveira et al., 2008). Thus, it was hypothesized that glycosylation pattern of *P. pastoris* inhibited this activity since non-glycosylated EcrFTL presented HA. In a previous work, *Pichia* glycosylation was also suggested to inhibit the HA of a fungal lectin (Iijima et al., 2003). Nevertheless, it should be noted that different FTL coding sequences were cloned in *P. pastoris* and *E. coli*, and hence the HA of the resulting proteins may differ. The deduced amino-acid sequences of the frutalin codifying genes cloned in *P. pastoris* and* E. coli* have 93% of sequence identity (**Figure 1**). In what concerns carbohydrate-binding activity, PprFTL presented a sugar preference similar to FTL, but with less affinity. The affinity constant for the binding of PprFTL to the monosaccharide methyl-α-galactose was determined and found to be 113-fold lower than that of FTL (Oliveira et al., 2008). The only other gJRL so far produced in microorganisms was jacalin. Jacalin was produced in *E. coli* also as an unprocessed protein with its sugar-binding activities reduced in the same order of magnitude (Sahasrabuddhe et al., 2004). The correct excision of the linker and consequent generation of a free glycine at the *N*-terminus of the α chain may determine gJRLs sugar-binding properties (Houles Astoul et al., 2002; Sahasrabuddhe et al., 2004; Oliveira et al., 2008).

The large-scale production of PprFTL was conducted in a 1.6 L stirred tank bioreactor operating in fed-batch mode at 28°C during 4 days (Wanderley et al., 2013). Supplementation of the culture medium (BMMH – buffered minimal methanol medium) with *Pichia* trace minerals (PTM) resulted in 2.5-fold higher PprFTL production (13.4 mg/l) than that achieved without supplementation (5.23 mg/l). Furthermore, bioreactor resulted in fourfold higher PprFTL production, comparing to shaker-flasks batch assays (3.3 mg/l), using the same culture medium (BMMH plus PTM) and induction conditions (Wanderley et al., 2013). Nevertheless, the yield of PprFTL was higher from shake-flasks induced at 15°C (18–20 mg/l), which means that lower temperatures favor PprFTL production (Oliveira et al., 2008). However, 20 of such flasks (each containing 50 ml of BMMH medium) are needed to obtain the same amount of PprFTL as in one fed-batch experiment. Thus, bioreactor fermentation is more advantageous for the production of PprFTL.

### BIOMEDICAL PROPERTIES

**FIGURE 2:**
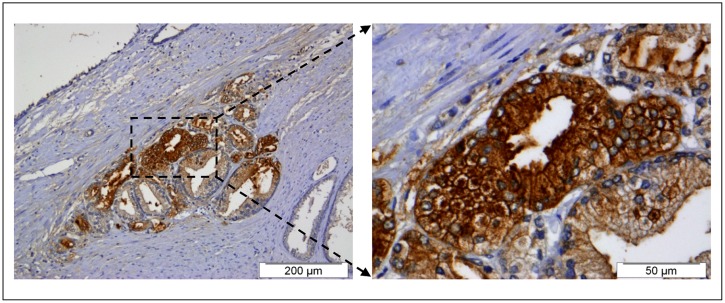
**Immunostaining pictures of a prostate cancer tissue using recombinant FTL produced in *P. pastoris* as tumor diagnostic marker.** PprFTL was able to specifically recognize carcinoma cells in middle of a benign lesion. The staining is localized in the cells cytoplasm of the carcinoma glands (brown color). (Original publisher: BioMed Central; Adapted from Oliveira et al., 2009b).

The relevance of JRLs, specifically jacalin, for cancer diagnostics and therapeutics is present in many recent works (e.g., Obaid et al., 2012; Lee et al., 2013; Marangoni et al., 2013; Zupancic et al., 2014). PprFTL was evaluated in terms of its tumor biomarker and anti-tumor properties, comparatively to FTL (Oliveira et al., 2009b, 2011). The cancer biomarker study was performed by immunohistochemistry with human prostate tissues (Oliveira et al., 2009b). Other plant lectins were used in the past in similar studies but with limited success (works cited in Oliveira et al., 2009b). The binding pattern of PprFTL and FTL to the prostate tissues was distinct, presumably due to their differences in carbohydrate-binding affinity (Oliveira et al., 2008). FTL bound to any type of prostate cells but more strongly to the neoplasic (malignant cells) than to the hyperplasic ones (non-malignant cells). On the other hand, PprFTL was much more specific, as it just recognized malignant cells (**Figure 2**). A significant positive statistical correlation between the binding intensity of PprFTL and the histological diagnosis of the tissues was obtained (not observed for FTL), although PprFTL did not recognize all the malignant cases studied (30% had negative binding), and when positive, the binding was heterogeneous. However, only a small number of prostate cases were analyzed and the histochemical methodology has still room for improvement. This study indicates that PprFTL has higher potential as cancer biomarker than FTL.

In *in vitro* assays, PprFTL showed a strong cytotoxic effect on HeLa cervical cancer cells proliferation, by inducing cell dead by apoptosis (Oliveira et al., 2011). This effect was irreversible as well as time and dose dependent (IC_50_ ∼ 100 μg/mL). Identical results were obtained for FTL in the same study (Oliveira et al., 2011). Thus, it seems that frutalin action in tumor cells is not exclusively dependent on its carbohydrate-binding properties. Other factors, such as protein–protein interactions, may contribute to the cellular responses, as suggested for the effect of recombinant jacalin in tumor cells (Sahasrabuddhe et al., 2006). Recombinant jacalin showed a magnitude of anti-proliferative responses similar to native jacalin on human cancer cell lines, despite its inferior sugar-binding affinity (Sahasrabuddhe et al., 2006). Both, PprFTL and FTL showed nuclear migration activity on HeLa cells (Oliveira et al., 2011), a property only reported for fungal lectins (Yu et al., 1999; Francis et al., 2003; Liang et al., 2009). Plant lectins have been described to attach to cancer cells membrane [e.g., jacalin in A431 human epidermoid carcinoma cells (Sahasrabuddhe et al., 2006)] or to be internalized and located in different cellular compartments [e.g., wheat germ agglutinin, WGA, in DU-145 human prostate cancer cells (Gabor et al., 2001)]. To our knowledge, our work is the first reporting nuclear migration activity on cancer cells for a lectin from plant origin (Oliveira et al., 2011). Studies aiming to elucidate the apoptotic mechanism triggered by PprFTL on cancer cells are now being conducted.

## CONCLUSION AND PROSPECTS

*Escherichia coli* and *Pichia pastoris* were found as suitable hosts for producing high amounts of recombinant FTL upon production and purification optimization. Optimization in *E. coli* significantly improved EcrFTL production, leading to high yields, but decreased protein stability. Furthermore, the processing of recombinant FTL in both microorganisms was different from that occurring in breadfruit, resulting in versions of FTL with inferior HA and carbohydrate-binding capacity. Nevertheless, PprFTL presented an anti-tumor activity identical to FTL and enhanced tumor biomarker capacity. The production strategies herein presented will extend the research on the biomedical properties of recombinant FTL.

The importance of amino-acids substitutions and post-translational modifications in gJRLs (e.g., linker cleavage, glycosylation) remains to be elucidated, and thus, future research might follow this direction. Within this scope, production and the availability of heterologous recombinant lectins is a valuable tool that can contribute to the fundamental understanding of the biological activity of the lectins. The production of the same lectin coding sequence in prokaryotic and eukaryotic hosts, the production of different lectin isoforms, and engineered/mutated versions, will provide insight into lectins functionality and shed light into its native physiological role. Furthermore, recombinant lectins with refined properties can be obtained. Finally, the fusion of enhanced lectins with functional moieties, by using recombinant DNA technology, for the development of functionalized drug delivery systems for site specific anti-tumor therapy, is anticipated.

## AUTHOR CONTRIBUTIONS

Carla Oliveira drafted the review and carried out most of the experimental work of recombinant FTL. José A. Teixeira participated in the development of the concept. Lucília Domingues conceived the study and helped to draft the review. All authors read and approved the final manuscript.

## Conflict of Interest Statement

The authors declare that the research was conducted in the absence of any commercial or financial relationships that could be construed as a potential conflict of interest.

## References

[B1] Al Atalah B. Fouquaert E. Vanderschaeghe D. Proost P. Balzarini J. Smith D. F. (2011). Expression analysis of the nucleocytoplasmic lectin ‘Orysata’ from rice in *Pichia pastoris*. *FEBS J.* 278 2064–2079 10.1111/j.1742-4658.2011.08122.x 21481190

[B2] Berlec A. Strukelj B. (2013). Current state and recent advances in biopharmaceutical production in *Escherichia coli*, yeasts and mammalian cells. *J. Ind. Microbiol. Biotechnol.* 40 257–274 10.1007/s10295-013-1235-0 23385853

[B3] Brando-Lima A. C. Saldanha-Gama R. F. Henriques M. Monteiro-Moreira A. C. Moreira R. A. Barja-Fidalgo C. (2005). Frutalin, a galactose-binding lectin, induces chemotaxis and rearrangement of actin cytoskeleton in human neutrophils: involvement of tyrosine kinase and phosphoinositide 3-kinase. *Toxicol. Appl. Pharmacol.* 208 145–154 10.1016/j.taap.2005.02.012 16183388

[B4] Brando-Lima A. C. Saldanha-Gama R. F. Pereira C. R. Villela C. G. Sampaio A. L. Monteiro-Moreira A. C. (2006). Involvement of phosphatidylinositol-3 kinase-Akt and nuclear factor kappa-B pathways in the effect of frutalin on human lymphocyte. *Int. Immunopharmacol.* 6 465–472 10.1016/j.intimp.2005.09.008 16428082

[B5] Campana P. T. Moraes D. I. Monteiro-Moreira A. C. Beltramini L. M. (2002). Unfolding and refolding studies of frutalin, a tetrameric D-galactose binding lectin. *Eur. J. Biochem.* 269 753–758 10.1046/j.0014-2956.2002.02742.x 11846776

[B6] Costa S. (2013). *Development of A Novel Fusion System for Recombinant Protein Production and Purification In Escherichia coli*. Ph.D. thesis, University of Minho, Braga

[B7] Costa S. Almeida A. Castro A. Domingues L. (2014). Fusion tags for protein solubility, purification and immunogenicity in *Escherichia coli*: the novel Fh8 system. *Front. Microbiol.* 5:63 10.3389/fmicb.2014.00063 PMC3928792 24600443

[B8] Costa S. J. Almeida A. Castro A. Domingues L. Besir H. (2013a). The novel Fh8 and H fusion partners for soluble protein expression in *Escherichia coli*: a comparison with the traditional gene fusion technology. *Appl. Microbiol. Biotechnol.* 97 6779–6791 10.1007/s00253-012-4559-1 23160981

[B9] Costa S. J. Coelho E. Franco L. Almeida A. Castro A. Domingues L. (2013b). The Fh8 tag: a fusion partner for simple and cost-effective protein purification in *Escherichia coli*. *Protein Expr. Purif.* 92 163–170 10.1016/j.pep.2013.09.013 24084009

[B10] de Vasconcellos Abdon A. P. Coelho De Souza G. Noronha Coelho De Souza L. Prado Vasconcelos R. Araujo Castro C. Moreira Guedes M. (2012). Gastroprotective potential of frutalin, a D-galactose binding lectin, against ethanol-induced gastric lesions. *Fitoterapia* 83 604–608 10.1016/j.fitote.2012.01.005 22285860

[B11] Fouquaert E. Smith D. F. Peumans W. J. Proost P. Balzarini J. Savvides S. N. (2009). Related lectins from snowdrop and maize differ in their carbohydrate-binding specificity. *Biochem. Biophys. Res. Commun.* 380 260–265 10.1016/j.bbrc.2009.01.048 19167365 PMC2681488

[B12] Francis F. Marty-Detraves C. Poincloux R. Baricault L. Fournier D. Paquereau L. (2003). Fungal lectin, XCL, is internalized via clathrin-dependent endocytosis and facilitates uptake of other molecules. *Eur. J. Cell Biol.* 82 515–522 10.1078/0171-9335-8 14629119

[B13] Fu L. L. Zhou C. C. Yao S. Yu J. Y. Liu B. Bao J. K. (2011). Plant lectins: targeting programmed cell death pathways as antitumor agents. *Int. J. Biochem. Cell Biol.* 43 1442–1449 10.1016/j.biocel.2011.07.004 21798364

[B14] Gabor F. Klausegger U. Wirth M. (2001). The interaction between wheat germ agglutinin and other plant lectins with prostate cancer cells Du-145. *Int. J. Pharm.* 221 35–47 10.1016/S0378-5173(01)00650-0 11397565

[B15] Gasser B. Prielhofer R. Marx H. Maurer M. Nocon J. Steiger M. (2013). *Pichia pastoris*: protein production host and model organism for biomedical research. *Future Microbiol.* 8 191–208 10.2217/fmb.12.133 23374125

[B16] Houles Astoul C. Peumans W. J. Van Damme E. J. Barre A. Bourne Y. Rouge P. (2002). The size, shape and specificity of the sugar-binding site of the jacalin-related lectins is profoundly affected by the proteolytic cleavage of the subunits. *Biochem. J.* 367(Pt 3), 817–824 10.1042/BJ20020856 12169094 PMC1222947

[B17] Iijima N. Amano K. Ando A. Nagata Y. (2003). Production of fruiting-body lectins of Pleurotus cornucopiae in methylotrophic yeast *Pichia pastoris*. *J. Biosci. Bioeng.* 95 416–418 10.1016/S1389-1723(03)80079-8 16233431

[B18] Lannoo N. Vervecken W. Proost P. Rouge P. Van Damme E. J. M. (2007). Expression of the nucleocytoplasmic tobacco lectin in the yeast *Pichia pastoris*. *Protein Expr. Purif.* 53 275–282 10.1016/j.pep.2007.01.007 17317217

[B19] Lee C. S. Muthusamy A. Abdul-Rahman P. S. Bhavanandan V. P. Hashim O. H. (2013). An improved lectin-based method for the detection of mucin-type O-glycans in biological samples. *Analyst* 138 3522–3529 10.1039/c3an36258b 23665615

[B20] Li C. Y. Luo P. Liu J. J. Wang E. Q. Li W. W. Ding Z. H. (2011). Recombinant expression of *Polygonatum cyrtonema* lectin with anti-viral, apoptosis-inducing activities and preliminary crystallization. *Process Biochem.* 46 533–542 10.1016/j.procbio.2010.10.005

[B21] Liang Y. Feng L. Tong X. Wang K. Li D. F. Lin J. C. (2009). Importance of nuclear localization for the apoptosis-induced activity of a fungal galectin AAL (Agrocybe aegerita lectin). *Biochem. Biophys. Res. Commun.* 386 437–442 10.1016/j.bbrc.2009.06.054 19527691

[B22] Liu B. Bian H. J. Bao J. K. (2010). Plant lectins: potential antineoplastic drugs from bench to clinic. *Cancer Lett.* 287 1–12 10.1016/j.canlet.2009.05.013 19487073

[B23] Marangoni V. S. Paino I. M. Zucolotto V. (2013). Synthesis and characterization of jacalin-gold nanoparticles conjugates as specific markers for cancer cells. *Colloids Surf. B Biointerfaces* 112 380–386 10.1016/j.colsurfb.2013.07.070 24028851

[B24] Mislovicova D. Gemeiner P. Kozarova A. Kozar T. (2009). Lectinomics I. Relevance of exogenous plant lectins in biomedical diagnostics. *Biologia* 64 1–19 10.2478/s11756-009-0029-3

[B25] Moreira R. A. Castelo-Branco C. C. Monteiro A. C. Tavares R. O. Beltramini L. M. (1998). Isolation and partial characterization of a lectin from *Artocarpus incisa* L. seeds. *Phytochemistry* 47 1183–1188 10.1016/S0031-9422(97)00753-X 9611823

[B26] Nobre T. M. Pavinatto F. J. Cominetti M. R. Selistre De-Araujo H. S. Zaniquelli M. E. Beltramini L. M. (2010). The specificity of frutalin lectin using biomembrane models. *Biochim. Biophys. Acta* 1798 1547–1555 10.1016/j.bbamem.2010.03.021 20353752

[B27] Obaid G. Chambrier I. Cook M. J. Russell D. A. (2012). Targeting the oncofetal Thomsen-Friedenreich disaccharide using jacalin-PEG phthalocyanine gold nanoparticles for photodynamic cancer therapy. *Angew. Chem. Int. Ed. Engl.* 51 6158–6162 10.1002/anie.201201468 22573473

[B28] Ohba H. Bakalova R. Muraki M. (2003). Cytoagglutination and cytotoxicity of Wheat Germ Agglutinin isolectins against normal lymphocytes and cultured leukemic cell lines – relationship between structure and biological activity. *Biochim. Biophys. Acta* 1619 144–150 10.1016/S0304-4165(02)00479-8 12527110

[B29] Oliveira C. Costa S. Teixeira J. A. Domingues L. (2009a). cDNA cloning and functional expression of the α-D-galactose-binding lectin frutalin in *Escherichia coli.* *Mol. Biotechnol.* 43 212–220 10.1007/s12033-009-9191-7 19521795

[B30] Oliveira C. Felix W. Moreira R. A. Teixeira J. A. Domingues L. (2008). Expression of frutalin, an α-D-galactose-binding jacalin-related lectin, in the yeast *Pichia pastoris*. *Protein Expr. Purif.* 60 188–193 10.1016/j.pep.2008.04.008 18534865

[B31] Oliveira C. Nicolau A. Teixeira J. A. Domingues L. (2011). Cytotoxic effects of native and recombinant frutalin, a plant galactose-binding lectin, on HeLa cervical cancer cells. *J. Biomed. Biotechnol.* 2011 568932 10.1155/2011/568932 PMC3206378 22131813

[B32] Oliveira C. Teixeira J. A. Domingues L. (2013). Recombinant lectins: an array of tailor-made glycan-interaction biosynthetic tools. *Crit. Rev. Biotechnol.* 33 66–80 10.3109/07388551.2012.670614 22530774

[B33] Oliveira C. Teixeira J. A. Schmitt F. Domingues L. (2009b). A comparative study of recombinant and native frutalin binding to human prostate tissues. *BMC Biotechnol.* 9:78 10.1186/1472-6750-9-78 PMC2754448 19740412

[B34] Pineau N. Pousset J. L. Preud’homme J. L. Aucouturier P. (1990). Structural and functional similarities of breadfruit seed lectin and jacalin. *Mol. Immunol.* 27 237–240 10.1016/0161-5890(90)90135-M 2111454

[B35] Raemaekers R. J. M. De Muro L. Gatehouse J. A. Fordham-Skelton A. P. (1999). Functional phytohemagglutinin (PHA) and *Galanthus nivalis* agglutinin (GNA) expressed in *Pichia pastoris* – Correct N-terminal processing and secretion of heterologous proteins expressed using the PHA-E signal peptide. *Eur. J. Biochem.* 265 394–403 10.1046/j.1432-1327.1999.00749.x 10491197

[B36] Sahasrabuddhe A. A. Ahmed N. Krishnasastry M. V. (2006). Stress-induced phosphorylation of caveolin-1 and p38 and down-regulation of EGFr and ERK by the dietary lectin jacalin in two human carcinoma cell lines. *Cell Stress Chaperones* 11 135–147 10.1379/CSC-160R.1 16817319 PMC1484514

[B37] Sahasrabuddhe A. A. Gaikwad S. M. Krishnasastry M. V. Khan M. I. (2004). Studies on recombinant single chain jacalin lectin reveal reduced affinity for saccharides despite normal folding like native jacalin. *Protein Sci.* 13 3264–3273 10.1110/ps.04968804 15557267 PMC2287297

[B38] Wanderley M. S. O. Oliveira C. Bruneska D. Domingues L. Lima J. L. Teixeira J. A. (2013). Influence of trace elements supplementation on the production of recombinant frutalin by *Pichia pastoris* KM71H in fed-batch process. *Chem. Pap.* 67 682–687 10.2478/s11696-013-0363-3

[B39] Yu L. G. Fernig D. G. White M. R. H. Spiller D. G. Appleton P. Evans R. C. (1999). Edible mushroom (*Agaricus bisporus*) lectin, which reversibly inhibits epithelial cell proliferation, blocks nuclear localization sequence-dependent nuclear protein import. *J. Biol. Chem.* 274 4890–4899 10.1074/jbc.274.8.4890 9988731

[B40] Zupancic D. Kreft M. E. Romih R. (2014). Selective binding of lectins to normal and neoplastic urothelium in rat and mouse bladder carcinogenesis models. *Protoplasma* 251 49–59 10.1007/s00709-013-0524-9 23828036

[B41] Zwierzina H. Bergmann L. Fiebig H. Aamdal S. Schoffski P. Witthohn K. (2011). The preclinical and clinical activity of aviscumine: a potential anticancer drug. *Eur. J. Cancer* 47 1450–1457 10.1016/j.ejca.2011.02.022 21482461

